# Clinical accuracy assessment of a dynamic navigation system and surgical guide using an oral appliance-secured patient tracker targeting anterior teeth

**DOI:** 10.1186/s40729-025-00627-5

**Published:** 2025-05-20

**Authors:** Manabu Okubo, Koudai Nagata, Yurie Okuhama, Kana Wakamori, Hayato Tsuruoka, Kurumi Saito, Ryota Fumimoto, Hiromasa Kawana, Shinji Kuroda

**Affiliations:** 1 Department of Regenerative Implant Dentistry, Kanagawa Dental University, 82 Inaoka-cho, Yokosuka, 238-8580 Japan; 2 Department of Oral and Maxillofacial Implantology, Kanagawa Dental University, 82 Inaoka- cho, Yokosuka, 238-8580 Japan

**Keywords:** Dental implant, Dynamic navigation system, Surgical guide, Guided surgery

## Abstract

**Purpose:**

Dynamic navigation systems and surgical guides have been reported to be equally accurate. However, the accuracy of dynamic navigation systems is affected by the movement of the patient tracker fixed to the tooth. We hypothesized that fixing the patient tracker to the oral appliance could improve accuracy. Therefore, this study aimed to compare accuracy between a dynamic navigation system with a patient tracker fixed to an oral appliance and a surgical guide.

**Methods:**

This observational study was conducted on patients who had undergone complete implant treatment at Kanagawa Dental University from 2020 to September 2024. Fifty implant bodies were placed in 42 patients with anterior tooth defects in both the dynamic navigation and surgical guide groups (25 implants each). DTX Studio™ (Nobel Biocare AG, Kloten, Switzerland) was used to overlay planning data on postoperative Digital Imaging and Communications in Medicine data to calculate entry point, apex point, and angular deviation accuracy.

**Results:**

The entry point, apex point, and angular deviation values were 0.99 ± 0.33 mm, 0.97 ± 0.43 mm, and 2.64 ± 0.87° in the dynamic navigation group and 1.33 ± 0.26 mm, 1.38 ± 0.3 mm, and 3.42 ± 1.03° in the surgical guide group, respectively, differing significantly at all measurement sites (*P* < 0.01).

**Conclusions:**

The fixation of the X-clip to the oral appliance improved intra-oral stability and inhibited intraoperative movement of the X-clip, resulting in high accuracy. These results suggest that dynamic navigation by oral appliance fixation is more accurate than surgical guides.

## Background

In implant-placement surgery, accurate positioning leads to optimal superstructure formation and helps prevent peri-implantitis during oral hygiene care [1, 2]. Dentistry is increasingly digitized; in the field of prosthetic-driven implant therapy, dynamic navigation systems and guided surgery using surgical guides are becoming popular [3, 4]. Compared to freehand surgery, guided surgery has improved the accuracy of implant placement and safety for the patient [5]. Dynamic navigation systems have been used in neurosurgery since their introduction in 2001, and these systems are now widely used in dentistry as well [6]. In Japan, the X-guide^®^ dynamic navigation system was launched in 2020. In the X-guide^®^, two cameras embedded in the illumination read and triangulate a hand-piece tracker and a patient tracker (X-clip) fixed to the patient’s teeth to provide real-time surgical support. However, the fixation of the X-clip to the tooth compromises its stability during mouth movement during surgery, directly affecting its accuracy. Therefore, fixing the X-clip in an oral appliance and attaching it to the dentition could improve its accuracy. In a previous study, we placed 25 implants in molar teeth using an X-clip fixed to the tooth and an X-clip fixed to an oral appliance and measured the accuracy. The entry point, apex point, and angular deviation values were 1.36 ± 0.51 mm, 1.30 ± 0.59 mm, and 3.20 ± 0.74°, respectively, with the X-clip fixed to the tooth, and 1.06 ± 0.31 mm, 1.02 ± 0.30 mm, and 2.91 ± 0.97°, respectively, with the X-clip fixed to the oral appliance, showing a significant difference between the entry and apex points in the two groups [7]. Fixing the X-clip to the oral appliance maintained stability, improved accuracy, and compensated for the shortcomings of the X-guide^®^.

In recent years, guided surgery using a surgical guide has been used for anterior teeth to improve esthetic outcomes. The disadvantages of the surgical guide include burns during bone formation, restricted openings in molars and other areas, and the need to perform surgery under indirect vision [8, 9]. In addition, surgical guides for anterior teeth are more prone to displacement than those for molars because of their reduced area of support [10]. However, dynamic navigation systems do not have these disadvantages. Therefore, we hypothesized that a dynamic navigation system might be better suited for anterior placement. Wang et al. reported similar accuracy for dynamic navigation systems and surgical guides, with measurements of 1.09 ± 0.41 mm and 0.83 ± 0.65 mm at the entry point, 1.55 ± 0.56 mm and 1.67 ± 0.94 mm at the apex point, and 3.37 ± 1.56° and 7.27 ± 3.82° for angular deviation, respectively. However, to our knowledge, no previous studies have compared the accuracy of dynamic navigation using a patient tracker fixed to an oral appliance with that of a surgical guide. Dynamic navigation using an oral appliance could offer better accuracy than a surgical guide [11]. Therefore, this study aimed to compare the accuracy of the X-guide^®^ with an oral appliance to that of a surgical guide for anterior teeth.

## Methods

**Fig. 1:**
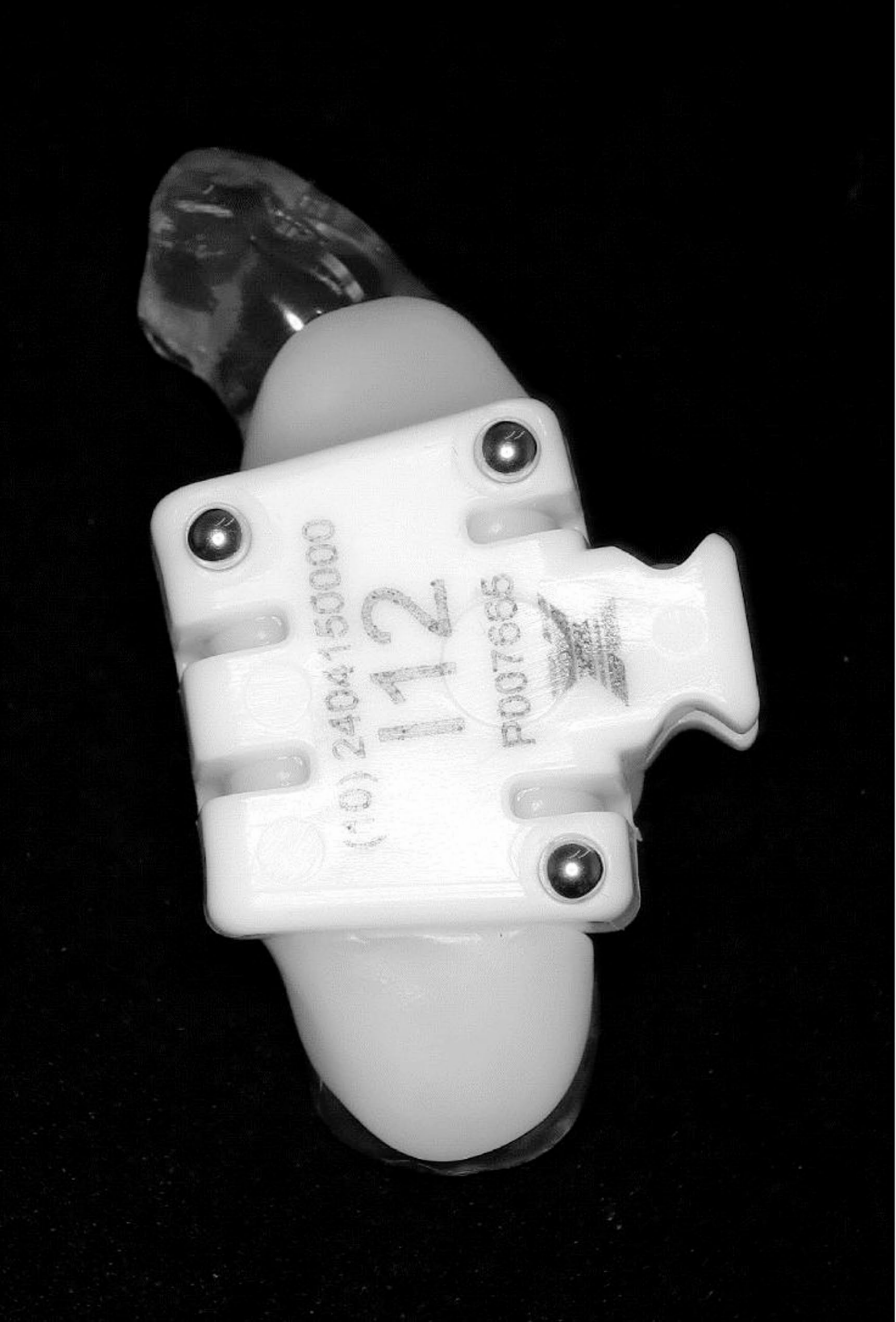
The patient tracker, X-clip, fixed to the oral appliance

This observational study was conducted on patients who had completed implant treatment at Kanagawa Dental University between April 2020 and September 2024. Forty-two partially edentulous patients (mean age, 56.0 years) with anterior tooth defects and 50 implant bodies were included in the study. Fifty implant bodies were placed in 21 patients in both the dynamic navigation and surgical guide groups. The inclusion criteria were patients ≥ 20 years of age with no more than three missing teeth and no concomitant bone-grafting procedures. The patients underwent optical impressions with an IntraOral Scanner (Trios^®^3, 3Shape, Copenhagen, Denmark) to obtain intraoral stereolithography (STL) data. The STL data were then loaded into computer-aided design (CAD) software (Exocad^®^; Exocad, Berlin, Germany) and the defects were digitally waxed up. Furthermore, the dynamic navigation group used CAD to determine the outline of the oral appliance based on the STL data. Specifically, the spacer was 0.05 mm, covering up to the maximum convexity of the cervical area of the unilateral dentition with the largest number of remaining teeth. After designing, a 1.2-mm-thick oral appliance was fabricated using a three-dimensional printer (Phrozen Sonic Mighty 4 K, Denken-Highdental Co., Ltd., Kyoto, Japan) and a DH Print Sprint & Guide (Denken-Highdental Co., Ltd.). Since X-clips are conventionally fixed to the teeth with no recommended method of fixing them to the oral appliance, the resin was immersed in boiling water to soften it until it became completely transparent. The resin was then fixed to the oral appliance and cured in cold water (Fig. 1). X-clips were fixed to an oral appliance, placed in the patient’s mouth, and images were recorded using cone beam computed tomography (CBCT; 3DX; Morita Co. Ltd., Tokyo, Japan). The surgical guide group was conventionally imaged with CBCT imaging and Digital Imaging and Communications in Medicine (DICOM) data were acquired. The imaging conditions were as follows: field of view, φ10 × H10; tube current, 5 mA; tube voltage, 89 kVP; slice thickness, 0.5 mm; and voxel size, 0.04 mm. All patient DICOM and STL data and digital wax-up data were loaded and superimposed in the planning software, DTX Studio™ (Nobel Biocare AG, Kloten, Switzerland) to select implant bodies and determine placement positions. The implant system used the implant bodies selectable in DTX Studio™. Specifically, 36 bone-level tapered implants φ3.3 mm (Straumann AG, Basel, Switzerland), six Nobel Replace conical connection narrow platform implants (Nobel Biocare AG), and eight NobelActive narrow platform implants (Nobel Biocare AG) were included. Table 1 shows the breakdown of the implant systems used in both groups.

**Table 1:** Breakdown of implant systems used in both groups

	Implant system	Number
Dynamic navigation group	Bone-level tapered implants (φ3.3 mm)	19
	Nobel Replace conical connection narrow platform implants	2
	NobelActive narrow platform implants	4
Surgical guide group	Bone-level tapered implants (φ3.3 mm)	17
	Nobel Replace conical connection narrow platform implants	4
	NobelActive narrow platform implants	4

**Fig. 2:**
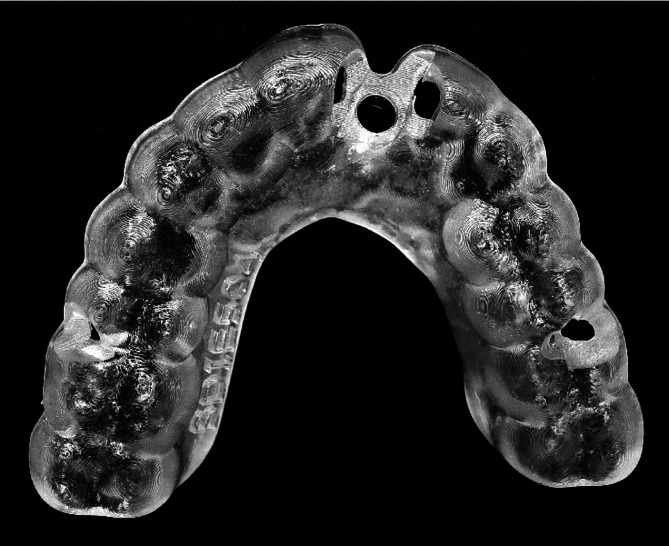
Surgical guide used as the fixation source for all remaining dentition

For the dynamic navigation group, planning data were exported and imported into the dynamic navigation system, X-guide^®^ (X-Nav Technologies, LLC, Lansdale, PA, USA). In the surgical guide group, after determining the placement position, the thickness of the surgical guide was designed to be 3 mm and to cover the entire remaining dentition (Fig. 2). The data were then sent to the manufacturer for modeling of the surgical guide. The Kanagawa Dental University Ethics Committee approved this study (approval number: 1045).

### Surgical procedure

All patients underwent infiltration anesthesia, gingival incision and avulsion, and surgical site preparation. In the dynamic navigation group, the hand-piece tracker and the patient tracker, X-clip, were calibrated preoperatively, and X-guide^®^ was used from surgical site preparation to implant placement. In the surgical guide group, Nobel Biocare AG implants were fully guided using a surgical guide until implant placement according to manufacturer’s instructions. Straumann AG implants were first drilled with Nobel Biocare AG 2 mm diameter drills with NobelGuide and then expanded with 2.4/2.8 mm diameter drills. Straumann AG drills were not used. The implants were then placed freehand using the half-guided method. All cases were performed in a two-stage implant placement, performed by three dentists under the supervision of a specialist accredited by the Japanese Society of Oral Implantology.

### Accuracy measurement

**Fig. 3:**
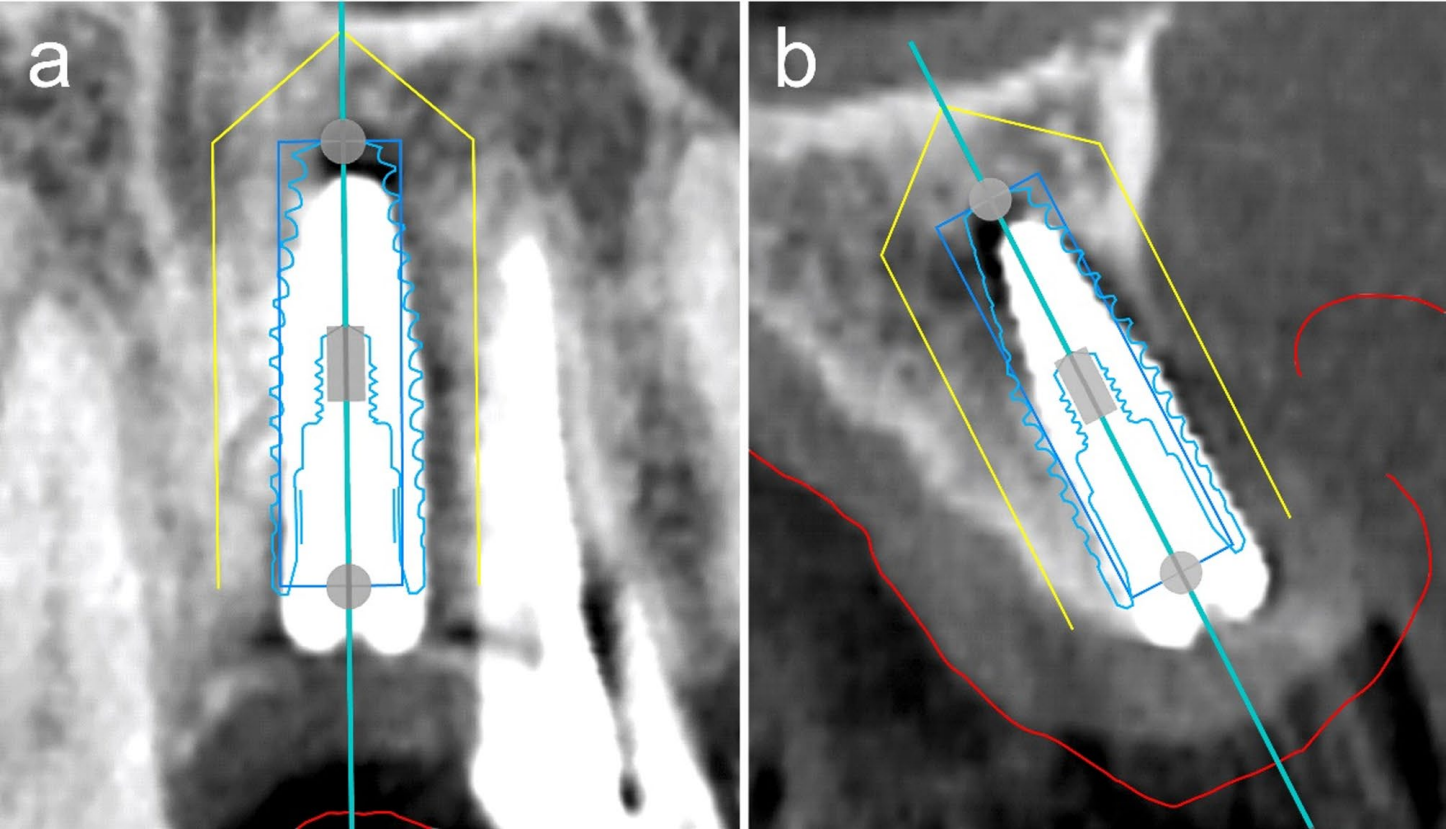
Superimposition of postoperative DICOM data and planning data. (**a**) coronal plane and (**b**) sagittal plane. DICOM, Digital Imaging and Communications in Medicine

**Fig. 4:**
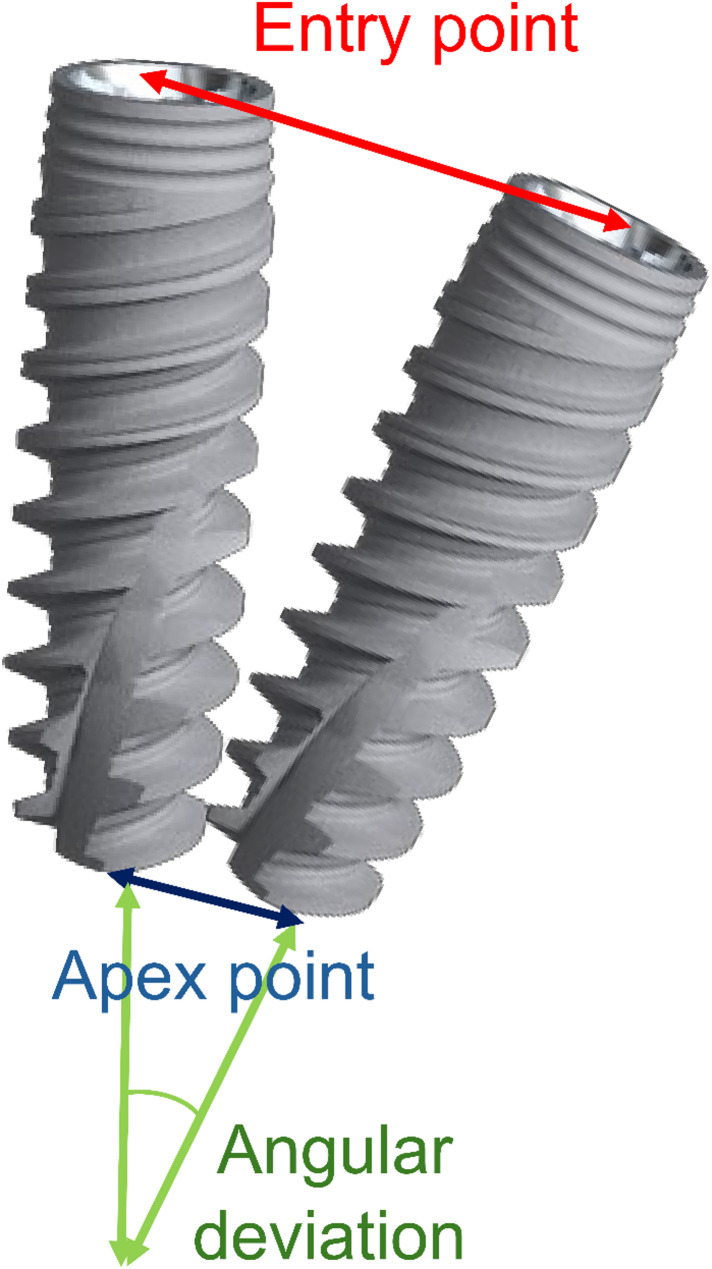
Entry points, apex points, and angular deviations. The entry points, apex points, and angular deviations were measured at two locations, parallel and perpendicular to the dentition

DTX Studio™ does not have an accuracy measurement tool. However, the preoperative planning data can be imported as STL into postoperative DICOM data, similar to the superimposition of intraoral STL data into preoperative DICOM data (Fig. 3). By superimposing the two datasets with respect to the remaining dentition, entry point, apex point, and angular deviation were measured at two locations, parallel and perpendicular to the dentition, and the average value was used as the result (Fig. 4). Further details have been listed in a previous study [7].

### Statistical methods

We used G-Power (version 3.1.9.2; Heinrich-Heine-Universität Düsseldorf, Düsseldorf, Germany) to calculate the required sample size. To obtain 80% power with an effect size of 0.8 at α = 0.05, the sample size was determined to be *N* = 25 [7]. Statistical analysis of each measurement site in both groups was performed using EZR (Saitama Medical Center, Jichi Medical University, Saitama, Japan). Normality was confirmed using the Kolmogorov–Smirnov test, and equal variance was confirmed using the F test [12]. Comparisons were then made using an unpaired t-test in BellCurve (Social Survey Research Information Corporation, Tokyo, Japan) for Excel. Results with *P* < 0.05 were considered significant.

## Results

The Kolmogorov–Smirnov test confirmed normally distributed data for entry point, apex point, and angular deviation results for both groups (all *P* > 0.05). An F-test performed to evaluate equivariance confirmed that the entry point, apex point, and angular deviation of both groups indicated the presence of equivariance (*P* < 0.05). The entry point, apex point, and angular deviation measurements were lower in the dynamic navigation group (0.99 ± 0.33 mm, 0.97 ± 0.43 mm, and 2.64 ± 0.87°, respectively) than in the surgical guide group (1.33 ± 0.26 mm, 1.38 ± 0.3 mm, and 3.42 ± 1.03°, respectively) (t-test: *P* < 0.001, *P* < 0.001, and *P* < 0.01 for entry point, apex point, and angular deviation, respectively).

## Discussion

In this study, the accuracy of a dynamic navigation system with X-clips fixed to an oral appliance and a surgical guide was measured and compared in patients with missing anterior teeth. Significant differences were observed in entry point, apex point, angular deviation, and all measurement sites, indicating the effectiveness of the dynamic navigation system using the oral appliance method for the anterior teeth.

The accuracy of dynamic navigation systems and surgical guides has been compared in several reports. A systematic review of 17 clinical studies by Yu et al. analyzed a total of 2,025 implants (dynamic navigation group: 1,526 implants and surgical guide group: 279 implants). The entry point, apex point, and angular deviation measurements were 1.07 mm, 1.27 mm, and 3.43°, respectively, in the dynamic navigation group. Compared to the surgical guide group, they reported an average difference of 0.02 mm for the entry point and − 0.07 mm for the apex point, which was not statistically significant [13]. Thus, the accuracy of the dynamic navigation system and the surgical guide has proven to be almost identical. Reports on the accuracy of implant placement for anterior teeth are scarce. Wu et al. compared the accuracy of 38 implants placed using a dynamic navigation system (Dental Implant Navigation System) with that of 57 implants placed using a surgical guide. The entry point, apex point, and angular deviation measurements were 1.36 ± 0.65 mm, 1.48 ± 0.65 mm, and 3.71 ± 1.32° in the dynamic navigation group and 1.22 ± 0.70 mm, 1.33 ± 0.73 mm, and 4.34 ± 2.22° in the surgical guide group, respectively. No statistically significant difference was observed between the two groups. Furthermore, the precision of placement of the anterior teeth for the entry point, apex point, and angular deviation in the dynamic navigation and surgical guide groups was 1.3 mm and 0.9 mm, 1.4 mm, and 1.1 mm, and 2.5° and 3.3°, respectively [14]. Younis et al. validated the accuracy of 94 implants in 65 patients. In the dynamic navigation group, the mean values across the 34 implants were 0.99 ± 0.52 mm for entry point, 1.14 ± 0.56 mm for apex point, and 3.66° ± 1.64° for angular deviation. They reported that the angular deviation of the molar implants was 4.12° ± 1.77° compared to 2.69° ± 0.67° for the anterior teeth, showing a significant difference [15]. Considering the above results, the accuracy of the dynamic navigation system and the surgical guide is comparable regardless of the site, and the dynamic navigation system can provide good angular deviation, especially in the anterior teeth. The accuracy of the surgical guide group in our study was similar to that in this report; however, the dynamic navigation group showed improved accuracy. This may be because fixing the X-clip in an oral appliance and attaching it to the dentition improved its stability in the oral cavity and prevented movement of the X-clip during surgery.

Several factors are involved in the accuracy of implant placement, including the planning software and the dynamic navigation system used [16]. Al-Ekrish measured the dimensional accuracy of implants using the planning software Blue Sky Plan, coDiagnostiX, and RadiAnt and reported no significant difference in error values between the software [17]. Wei et al. measured the implant-placement accuracy of ImplaNav, IRIS, X-Guide, NaviDent, and AqNavi based on 10 articles and reported no difference among the five dynamic navigation systems [18]. Yu et al. also compared the accuracy of IRIS-100, DCarer, ImplaNav, X-Guide, AqNavi, and Navident and reported that the six dynamic navigation systems showed a significant difference (*P* = 0.03) in entry point, but not for apex point and angular deviation [13]. Based on these reports, any of the planning software and dynamic navigation systems used are almost equally accurate. Ma et al. defined the accuracy of the dynamic navigation system depending on the size and length of the implant, although three implant systems were used in their study. The accuracy of implants of 3.5, 4.3, and 5 mm in diameter (*P* = 0.32 for entry point, *P* = 0.76 for apex point, and *P* = 0.4 for angular deviation) were compared and no significant difference was found. They also compared the accuracy of implants with lengths of 8.5, 10, 11.5, and 13 mm and reported no significant differences (*P* = 0.55 for entry point, *P* = 0.5 for apex point, and *P* = 0.59 for angular deviation) [19]. Therefore, the implant system does not interfere with the accuracy of dynamic navigation. Based on these studies, the most important factor regarding accuracy may be the measurement method. Examples of planning software that can measure the accuracy of implants after placement include coDiagnostiX and DentiqGuide. coDiagnostiX can measure accuracy by superimposing intraoral STL data equipped with a scan body on the planning data [20]. DentiqGuide superimposes postoperative DICOM data on the planning data, allowing for measurement of accuracy [21]. However, accuracy measurement tools do not exist in many planning software programs. Therefore, preoperative DICOM data, planning data, and postoperative DICOM data are often exported and imported into an editing software to measure accuracy [18]. However, these processes are complex and errors due to the process and artifacts can adversely affect accuracy [22]. To our knowledge, no studies have reported accuracy measurements using DTX Studio™ other than our previous study and the present report. The efficacy of these methods of measuring accuracy needs to be assessed.

Regarding the accuracy of the surgical guide, Kholy et al. reported that the extent of the surgical guide affects the accuracy of the implant placement. For a single anterior tooth defect, on using the entire remaining dentition as the fixation source, the entry point, exit point, and angular deviation were of 0.28 mm, 0.68 mm, and 4.36°, respectively, and on using two teeth on either side of the defect as the fixation source for the surgical guide, the entry point, exit point, and angular deviation were of 0.289 mm, 0.62 mm, and 4.73°, respectively, with no significant difference [10]. However, considering the limited reports on the extent of the surgical guide and the lack of evidence, this study was designed to cover the entire remaining dentition. Chen et al. compared the accuracy of a full guide, which uses a surgical guide up to the placement of the implant, and a half guide, which uses a surgical guide only for surgical site preparation, with the placement of the implant performed freehand. For the full guide, the entry point, exit point, and angular deviation were of 0.53 ± 0.29 mm, 1.10 ± 0.42 mm, and 2.09 ± 1.07°, respectively. For the half guide, the entry point, exit point, and angular deviation were of 0.94 ± 0.43 mm, 1.51 ± 0.55 mm, and 3.06 ± 1.92°, respectively, reporting significant differences in apex point and angular deviation [23]. Their half-guide method was the method of placing Nobel Biocare AG implant bodies using a drill system from another company. This method was similar to our surgical guide group’s method of placing Straumann AG implants using the Nobel guide. The inability to adjust the depth at the time of placement in the half guide is considered to be the reason for its lower accuracy than the full guide. In the present study, we used three different implant systems in the surgical guide group, and for the Straumann AG implants, we used a half guide because of the difference in the form of the surgical guide sleeve. This may have affected the accuracy of the surgical guide group. However, the dynamic navigation system with X-clips fixed to the oral appliance led to improved accuracy in the anterior teeth.

A limitation of this study is that identical implant systems could not be used. The accuracy of the surgical guide group would be improved if identical implant systems could be used. In the future, an identical implant system should be used to compare accuracy.

## Conclusions

In this study, the accuracy of a dynamic navigation system with X-clips fixed to an oral appliance and a surgical guide was measured and compared in patients with missing anterior teeth. Significant differences were observed for entry point, apex point, and angular deviation, indicating the effectiveness of the dynamic navigation system using the oral appliance method for the anterior teeth. Dynamic navigation systems have fewer drawbacks than surgical guides, but intraoperative patient tracker upheaval poses concerns. The fixation of the X-clip to the oral appliance improved intra-oral stability and inhibited intraoperative movement of the X-clip, which is considered to have resulted in good accuracy.

## Data Availability

The datasets used and/or analysed during the current study are available from the corresponding author on reasonable request.

## Abbreviations

CAD: Computer-aided design
CBCT: Cone beam computed tomography
DICOM: Digital imaging and communications in medicine
STL: Stereolithography
